# Health outcome convergence and the roles of public health financing and governance in Africa

**DOI:** 10.1371/journal.pone.0312089

**Published:** 2024-10-15

**Authors:** Ariane Ephemia Ndzignat Mouteyica, Nicholas Nwanyek Ngepah

**Affiliations:** School of Economics, College of Business and Economics, University of Johannesburg, Johannesburg, South Africa; Universitatsklinikum Schleswig Holstein Campus Lubeck, GERMANY

## Abstract

Progress in health outcomes across Africa has been uneven, marked by significant disparities among countries, which not only challenges the global health security but impede progress towards achieving the United Nations’ Sustainable Development Goals 3 and 10 (SDG 3 and SDG 10) and Universal Health Coverage (UHC). This paper examines the progress of African countries in reducing intra-country health outcome disparities between 2000 and 2019. In other words, the paper investigates the convergence hypothesis in health outcome using a panel data from 40 African countries. Data were sourced from the World Development Indicators, the World Governance Indicators, and the World Health Organization database. Employing a non-linear dynamic factor model, the study focused on three health outcomes: infant mortality rate, under-5 mortality rate, and life expectancy at birth. The findings indicate that while the hypothesis of convergence is not supported for the selected countries, evidence of convergence clubs is observed for the three health outcome variables. The paper further examine the factors contributing to club formation by using the marginal effects of the ordered logit regression model. The findings indicate that the overall impact of the control variables aligns with existing research. Moreover, governance quality and domestic government health expenditure emerge as significant determinants influencing the probability of membership in specific clubs for the child mortality rate models. In the life expectancy model, governance quality significantly drives club formation. The results suggest that there is a need for common health policies for the different convergence clubs, while country-specific policies should be implemented for the divergent countries. For instance, policies and strategies promoting health prioritization in national budget allocation and reallocation should be encouraged within each final club. Efforts to promote good governance policies by emphasizing anti-corruption measures and government effectiveness should also be encouraged. Moreover, there is a need to implement regional monitoring mechanisms to ensure progress in meeting health commitments, while prioritizing urbanization plans in countries with poorer health outcomes to enhance sanitation access.

## 1. Introduction

Over the past two decades, there has been a remarkable transformation in the global health landscape, leading to longer and healthier lives for people worldwide. This improvement is attributed to better national, regional, and international health policies and the effective use of development assistance [1]. Moreover, advancements in health services and sanitation facilities have contributed to declining mortality rates [2]. In addition, the free flow of technology, individuals, and production techniques has also played a critical role in influencing health outcomes. However, challenges in providing adequate health financing, human resources, and equitable healthcare service delivery remain crucial issues that must be addressed [3]. In Africa, there have been significant improvements in health over the years. Between 1970 and 2010, the under-five mortality rate (UMR) declined by over 50 percent. Additionally, from 1990 to 2010, infant mortality decreased from 99 to 71 deaths per 1,000 live births. Life expectancy at birth also rose from 38 years in 1950 to 56 years in 2012 [4]. There was also a decrease in the number of new HIV/AIDS infections and AIDS-related deaths. Moreover, several preventive measures, such as the distribution of free insecticide-treated bed nets significantly decreased malaria incidence and mortality rates [4].

The African Union together with regional economic communities (RECs) have demonstrated their commitments in implementing several policies and frameworks that encompass various continental and global commitments and strategies in the health sector that aim at guiding, inspiring, and highlighting strategic direction useful for all the African Union member states [5]. These initiatives include the Abuja Declaration in 2001, the Maputo Plan of Action and campaigns, including the Campaign for Accelerated Reduction of Maternal Mortality in Africa (CARMMA), numerous Ministerial and AU Assembly decisions and declarations, African Health Strategy 2007–2015 and the African Health Strategy 2016–2030, among others. Some of these initiatives were developed in the context of the MDGs era, whereas others were developed following the “2063 Agenda: The Africa We Want” and the SDGs of the 2030 Agenda for the UN Sustainable Development. Additionally, these initiatives provide more comprehensive, actionable, and flexible platform that can enable African countries to converge and align to improve coherence and synergy in improving health outcomes in the region [5].

A significant amount of literature has shown that regional integration creates forces for convergence among countries [6–8]. For instance, regional integration entails a common approach to policy formulation, leading to commonality in terms of economic performance, institutions, regulation, access to infrastructure, and policymaking processes [9]. Additionally, previous studies have shown that the main drivers of convergence in health expenditure include integration of health care markets and common policies that promote health, good working condition, coordination of medical research, insurance coverage, and the diffusion of healthcare technologies and products [8, 10].

Despite its advances in recent years, the area of African health and regionalism remains beset by multiple knowledge gaps, both empirical and theoretical in nature. Empirical and systematic research about how African health is impacted by regional integration has attracted less attention among health economists, especially outside Southern and Western Africa. Health remains far less scrutinised, despite the large number of studies on many social sectors in the literature. Focusing on health as a specific social sector, particularly in the African context, is important, given that access to healthcare for all Africans is still a significant challenge for policymakers. The continent has the lowest level of health expenditures, while accounting for 24 percent of the global disease burden and 11 percent of the global population. Africa is also plagued by high levels of poverty and a heavy burden of infectious and non-infectious diseases, which are major cause of mortality and morbidity. Some of these diseases are preventable [11]. Thus, a better understanding of the impact of regional integration on health will be significant in assessing whether health policies at the regional and continental levels reduces disparities in health across member states, leading to convergence over time.

Focusing on convergence in health outcomes in the context of regionalism has several policy implications. Promoting convergence in health outcomes among African countries is important, particularly in the context of pandemics and epidemics, despite deteriorating macroeconomic conditions. In this study, convergence implies narrowing the gaps in health outcomes. This is vital in attaining regional stability, economic development, and global health security [12–15]. Sustainable strategies should be implemented, even during macroeconomic challenges like the COVID-19 pandemic, to ensure the long-term well-being of the continent. Furthermore, health outcome convergence studies are essential to evaluate the effectiveness of the existing health policies that aim at minimizing cross-country health outcome disparities [12]. Moreover, academic research and evidence play a significant role in guiding new policies and interventions aimed at improving the health and well-being of populations. Monitoring convergence in health outcomes in Africa is also crucial for tracking progress towards achieving global, continental, and regional health goals, including the SDGs and UHC [13, 14]. Furthermore, health outcome convergence studies are significant tools that can empower civil society organizations and advocates to hold governments accountable for health outcomes. Additionally, the identification of the factors contributing to health outcome convergence can stimulate research and innovation in healthcare systems and interventions tailored to the specific needs of different countries or sub-group of countries [15, 16].

There are theoretical and empirical evidence regarding the impact of regional integration on health convergence. However, most of these studies focus on the OECD and EU region [17, 18]. Only a few studies have focused on Africa, particularly in the context of regional integration. Thus, in this study, we will see whether common policies at regional and continental levels have been effective in bring about convergence in health outcomes in the continent. This study attempts to answer the following question: Are the disparities in health outcomes between African countries narrowing or widening over time? To address this question, we chose three health outcome indicators: the infant mortality rate (IMR), the under-five mortality rate (U5MR), and the life expectancy at birth (LEB). We use a non-linear dynamic factor model on a panel of 40 African countries. The methodology is based on a non-linear time-varying factor model that incorporates the possibility of transitional heterogeneity or even transitional divergence. The method also allows the identification of possible convergence clubs in the sample [19, 20]. This has significant policy implications, including addressing disparities, advancing global and regional health goals, and achieving health equity [14–16].

Furthermore, we use the marginal effects of the ordered logit regression model to examine the factors that determine club formation. We also use the principal component analysis (PCA) to construct the quality of governance index. This index will allow us to assess the effects of governance quality on club formation. Previous studies showed that governance is a significant determinant of health outcomes, given its functions in implementing health policies and influencing socioeconomic conditions within and between countries [21]. We also consider the effects of the share of domestic general government health expenditure to general government health expenditure. This indicator is mainly seen as the proxy of the 2001 Abuja Declaration on allocating at least 15 percent of national budgets to health. It also shows the level of government prioritization of the health sector [22].

This paper is structured as follows: Section 2 explains the literature and empirical review, Section 3 describes the empirical methods used in the study, Section 4 presents the results, Section 5 provides the discussion of the results, and Section 6 provides the conclusion and policy recommendations.

## 2. Theoretical and empirical review on health outcome convergence

The question of whether poorer countries catch up to the rich ones has received significant attention in growth theory and development economics. Convergence, defined as a process of equalization or uniformity of levels of development among countries, is a crucial condition for efficient and successful integration [17]. It originated from the neoclassical growth model. It was used to explain the trends in income per capita, i.e. economic disparities arising from integration to international trade activities, capital accumulation, labour, and capital productivity [23]. Broadly speaking, convergence suggests that capital will flow from capital abundant countries to countries with low capital formation, leading to the latter catching up with the former. This is because the capital recipient countries would experience higher productivity. In this context, globalisation and regionalism become crucial because they do not only facilitate the flow of capital, but also goods, technology, human capital, necessary for convergence [12, 24].

Convergence hypothesis has been applied to various economic indicators, including military expenditure, health expenditure, and health outcomes [12, 25, 26]. The neoclassical growth theory identifies four methods to test the convergence hypothesis: beta, sigma, stochastic, and club convergence. Beta convergence suggests that countries with low initial average income per capita tend to grow faster and catchup with countries with high average income per capita, due to decreasing diminishing return to capital [27, 28]. If the correlation between the growth rates of an economic variable and its initial level is negative and significant, beta convergence is evident, while a positive correlation suggests a divergence behaviour of low-income countries. Sigma convergence measures the cross-sectional dispersion. Convergence occurs when the variations of an economic variable between countries reduces over time [29].

However, these convergence approaches have many limitations. For instance, some studies revealed that the results of β-convergence may be biased and inconsistent, due to the assumption of equal speed of convergence, homogeneity in technological progress, and initial income between countries [30, 31]. Additionally, the unobserved heterogeneity between countries affects the empirical estimations as a result of endogeneity and omitted variable issues. The β-convergence collapses due to stochastic technological progress [32]. On the other hand, σ-convergence provides the necessary, but not sufficient, way for observing the decrease dispersion of income per capita. The dispersion of a given variable should disappear in the long run if countries with similar technologies and structural characteristics convergence towards a common equilibrium level. If, however, countries converge to clubs or their own unique equilibrium, the dispersion of real per capita income will not reach zero [33, 34]. Additionally, rejecting the sigma convergence hypothesis does not necessarily imply divergence across countries, because transitional dynamics in the data may cause the rejection of the null hypothesis of sigma convergence [17]. Stochastic convergence was therefore introduced to account for stationarity issues. This occurs when a country’s economic variable relative to the benchmark country is stationary, revealing a steady state level of the economic variable. For this reason, stochastic convergence suggests that long-run growth does not depend on idiosyncratic country-specific factors. Additionally, the differences between countries are not persistent, leading to converge in the mean zero stationary processes [35].

However, studies have shown that in the absence of a structural break or cyclical components, stochastic convergence becomes sensitive to less effective univariate tests and may lead to misspecification errors and biased results [36]. Although revisited by growth analysist in the mid-1980s, the convergence club approach gained prominence in the 2000s with the introduction of the non-linear time-varying factor model, which accounts for individual and transitional heterogeneity of countries [19, 33, 37, 38]. In recent years, some studies have used the non-linear time-varying approach to investigate the convergence club hypothesis among European and OECD countries. They also introduced the concept of club merging, which allows for the merging between the initial clusters, when the clustering procedure overestimates the number of initial clubs [20, 39].

Furthermore, criticisms of the neoclassical growth model have shown that there are differences in growth over time, particularly in large samples of countries, even among developing countries. Endogenous growth models, recently developed, can generate sustained growth without resorting to exogenous technological progress. Endogenous growth models predict overall divergence in income levels ad growth rates. The theory suggests that the rate of technological progress is determined endogenously by decisions of individual countries to invest in human or physical capital, persistent disparities in growth rates across countries can arise due to variations in rates of accumulation of factor endowments. However, in the case of exogenous technological change, the lack of decrease in the marginal productivity may lead to divergence and sustained growth due to the accumulation of capital [12]. In theoretical models, the assumptions of the endogenous growth theory are dependent on the character of technology and market interactions. Thus, the presence of divergence contradicts the neoclassical growth model predictions, where complete competitive markets under a constant return technology provides decreasing return to investment, thereby yielding slowing and later ceasing growth [17]. However, this paper focuses on the neoclassical growth model. Although there is no absolute homogeneity in terms of technology in African countries at the continental and regional levels, most countries list amongst developing countries, and thus, fall within the convergence expectations in the neoclassical convergence theory.

Several studies have investigated the convergence hypotheses in health, with mixed results. For instance, some empirical studies showed that countries with low mortality experienced less gains in longevity compared to countries with high mortality. Consequently, the death rate in high mortality countries would fall significantly over time, leading to worldwide convergence in mortality [40, 41]. However, other studies revealed that the gains in life expectancy at birth do not necessarily translate into convergence in population health status [42]. In the EU region, a study found divergence in health outcomes among 15 countries between 1980 and 1995, whereas there was σ-convergence in per capita health expenditures across the countries. The authors also showed evidence of absolute and conditional in health spending, suggesting that convergence analysis in health expenditure supports effort for deeper EU integration. They also revealed that GDP per capita, service coverage, and population structure significantly affect convergence among EU countries [17]. In contrast, evidence of β−and σ−convergence in life expectancies was found across the EU countries [43]. In Africa, there was evidence of β-convergence in health outcomes in the ECOWAS region [44]. A similar another study was done for the region, with the same results. While the authors’ results did not support the hypothesis of σ−convergence in life expectancy, the showed that trade openness and governance were the main drivers of convergence in ECOWAS [45].

In many countries and regions, the process of demographic and epidemiological transition has led to a more unequal distribution of health outcomes over time, significantly affecting the between and within country health outcome inequalities [40]. This is in part due to the combination of socioeconomic and political challenges, including unequal access to basic healthcare services, conflict and war, climate change, rising number of older people, socioeconomic status disparities, and low investments in health [46, 47]. For instance, some studies revealed that governments low health financing, low investments in sectors such as water, sanitation, food security, and housing, as well as high reliance on out-of-pocket spending and inefficient health programme integration have negative impact on life expectancy at birth among ECOWAS countries, whereas their effects on childhood mortality are positive [22]. Furthermore, political democratization, economic development, and nutritional improvements have a long-term positive effect on life expectancies [48].

A study examining the impact of economic development on life expectancy among 65 countries along the B&R from 2000 to 2014 found that the effect of macroeconomic factors on life expectancy depends on its distribution. While unemployment rate was positively associated with life expectancy for the countries in the top life expectancy quantiles, GDP growth and inflation had a negative impact on life expectancy in the bottom life expectancy quantiles for men only [49]. Additionally, factors such as health spending, food supply, and urbanization improve life expectancy in Pakistan, while illiteracy and increased economic misery reduce it [50]. Recent evidence in Bangladesh showed that increased health expenditure, skilled delivery attendance, immunization coverage, and improved healthcare provisions tend to reduce mortalities among children and infants [51]. Similarly, public health spending as a share of GDP, urbanization, and GDP per capita negatively affect infant mortality rate among WAEMU countries [52].

Inasmuch, the discussion on convergence in health outcomes has not yet been fully explored from a theoretical and methodological perspective. Given the significance of this question, a few studies have attempted to investigate the issue, but most have focused on OECD and European countries. In Africa, there is only a few evidence-based testing of convergence models for health outcome. Furthermore, hardly any study has explored the non-linear time-varying method [19, 20]. This method has several advantages over the conventional β-convergence, σ-convergence, and stochastic convergence approaches. It is not subjected to any strict assumption of variable stationarity and common factors. It accounts for changing behaviour of countries, and enables the detection of convergence clusters between heterogeneous countries. It also has the capacity to detect club merging between the initial clusters, when there is an overestimation of the real number of initial clubs [25, 26]. The club convergence approach is also an important tool to assess the performance of countries over time. The study also uses the marginal effects of the ordered logit regression to identify the determinants of club formation, which is crucial for guiding health policies and intervention, advancing health goals at the national, regional, and global levels, addressing health inequalities, and achieving health equity and health-related SDGs [16].

## 3. Methodology

### 3.1 Log-t test

The theoretical framework of this study derives from the neoclassical growth literature. We use the log *t* test, the clustering algorithm, and merging club algorithm. The log *t* test is based on the decomposition of the variables of interest into two components as follows:

$$y_{it}=\rho_{it}+\epsilon_{it}$$ (1)

Where *ρ*_*it*_ is a systematic component and *ϵ*_*it*_ is a transitory component. To separate the common from idiosyncratic components, [19] further transformed Eq (1) as follows:

$$y_{it}=(\frac{\rho_{it}+\epsilon_{it}}{\sigma_{t}})\sigma_{t}=\delta_{it}\mu_{t},\;for\;all\;i,t$$ (2)

Where *y*_*it*_ is decomposed into two time-varying components: the common component *σ*_*t*_ and the idiosyncratic component *δ*_*it*_. The latter measures the distance between *y*_*it*_ and the common component *σ*_*t*_, while absorbing the error term and the unit-specific component and varies over time. This formulation allows us to test whether the factor loadings *δ*_*it*_ converge to a constant δ, by taking ratios instead of differences and eliminating the common component.

The following semi-parametric specification to characterize the dynamics of the idiosyncratic component was further proposed [19].

$$\delta_{it}=\delta_{i}+\frac{\theta_{i}\xi_{it}}{{L(t)t}^{a}}$$ (3)

Where *δ*_*i*_ is fixed, *ξ*_*it*_ is *iid* (0, 1) across *i*, *θ*_*i*_ are idiosyncratic scale parameters, *L*(*t*) is a slowly varying function, such as log(*t*), so that *L*(*t*)⇾∞ as *t*⇾∞. The parameter *a* represents the speed of convergence. This formulation ensures that *δ*_*it*_ converges to δ for all *a*≥0.

Estimating of the time-varying factor loadings *δ*_*it*_ contains information about the transition behavior of particular panel units, which are using the relative transaction parameter defined as:

$$h_{it}=\frac{y_{it}}{\frac{1}{N}\sum_{i=1}^{N}y_{it}}=\frac{\delta_{it}}{\frac{1}{N}\sum_{i=1}^{N}\delta_{it}}$$ (4)

Where *h*_*it*_ measures the relative departure of country *i* from the common steady-state growth path *μ*_*t*_, therefore, divergence from *μ*_*t*_ is reflected from the transition path *h*_*it*_. The cross-sectional mean of the relative transition path *h*_*it*_ is unity. When panel units converge and the factor loadings *δ*_*it*_ approach to a constant δ, the relative transaction path *h*_*it*_ converges to unity, and the cross-sectional variation *V*_*t*_ of the relative transition path converges to zero as *t*→∞, as follows:

$$V_{t}=\frac{1}{N}\sum_{i=1}^{N}(h_{it}-{1)}^{2}\to 0,as\;t\to \infty$$ (5)

[19] also proposed a convergence test and clustering algorithm based on the log *t*-test. The latter is based on a simple time series regression involving a one-sided t-test. The null hypothesis is as follows:

*H*_0_: Convergence for all *i H*_0_: *δ*_*i*_ = δ and ≥0: convergence for all member states, while the alternative is: *H*_*A*_: No convergence for all *i H*_*A*_: *δ*_*i*_ ≠ δ and <0: divergence for some member states. Finally, the null hypothesis of convergence is tested based on the following log *t* regression:

$$\log\left(\frac{V_{1}}{V_{t}}\right)-2logL(t)=\hat{c}+\hat{b}logt+\mu_{t},for\;t=[rT],[rT]+1\ldots ,T$$ (6)

Where *V*_*t*_ is the cross-sectional variation, $\frac{V_{1}}{V_{t}}$ is the ratio of the cross-sectional at the beginning of the sample *V*_1_ (i.e. *V*_*t*_ at t = 1) over the respective variation for every point in time t, that is *V*_*t*_ (t,…,T), *L*(*t*) is log (*t*) and *r*>0. Based on Monte Carlo experiments, [19] suggest setting *r* equal to 0.3 when T ≤ 50. The null hypothesis of convergence is supported if *t*_*b*_>−1.65. The standard error estimates are calculated using a HAC estimator for the long-run variance of the residuals.

### 3.2 Club convergence algorithm

The rejection of the null hypothesis does not necessarily mean that there is an absence of convergence of subgroups in the panel. Thus, we will use the empirical algorithm to test for convergence clubs.

1. Sorting countries in the sample in decreasing order based on their observations in the last period.
2. Identifying a core group of *K* countries where *K*<*N*, for which the log *t* result has the highest t-statistics.
3. Adding one country at a time from the remaining countries to the core group and applying the log *t* test. The newly added country satisfies the membership condition if *t*_*b*_>−1.65.
4. Repeating steps 1–3 on all the remaining countries to identify other possible convergence clubs.

To avoid overestimating the initial number of clubs that may arise from using a sign criterion in step 2, [20] introduced the club merging test, which suggests that if *t*_*b*_> -1.65, the initial clubs are merged at the five percent significance level.

### 3.3 Ordered logit model

While the clustering methods advocated by [19, 20] can identify convergence clubs, they do not offer insights into the factors influencing club formation. Therefore, this study endeavors to uncover the underlying factors responsible for club formation and quantify their impacts across the identified clubs. To do so, we employed ordered logit regression model. This is because the final convergence clubs resulting from the PS procedure are structured as ordinal variables with meaningful order [19, 20]. This method offers a way to evaluate ordering information, unlike multinomial logistic regression, which often overlooks the ordered nature of the convergence clubs. The ordered logit model has been applied across diverse fields [53].

Let *D*_*i*_ be the ordinal response variable and *C* the countries belonging to the convergence clubs. *H*_*i*_ represent the vectors of explanatory variables influencing club formation and *U*_*ic*_ denote the probability that a country *i* belongs to the convergence club *c*, which is the dependent variable. Eq (7) shows the cumulative probabilities if the convergence clubs are arranged sequentially, such as: *c* = 1,…,*C*.


$$G_{ic}=A(Y_{i}\leq \frac{Y_{c}}{Q_{i}})$$ (7)

The ordered logit regression model establishes a connection between the independent variables and the likelihood of the convergence clubs *C*. This link is articulated through a series of *C*−1 equations derived from cumulative probabilities, outlined as follows:

$$\log(\frac{G_{ic}}{1-G_{ic}})=\varepsilon_{c}-\alpha H_{i},c=1,2,\ldots \ldots ,C-1$$ (8)

Where the total count of independent variables is represented as $\alpha_{1}H_{i1}+\alpha_{2}H_{i2}+\cdots {+\alpha }_{n}H_{in}$. The probability of membership in a specific convergence club is determined by the mean value of *H*_*i*_ and is depicted as follows:

$$p\left(\gamma =\frac{1}{H_{i}}\right)=\frac{\exp\left(\varepsilon_{c}-\alpha H_{i}\right)}{1+\exp\left(\varepsilon_{c}-\alpha H_{i}\right)},for\;c=1$$ (9)

$$p\left(\gamma =\frac{c}{H_{i}}\right)=\frac{\exp\left(\varepsilon_{c}-\alpha H_{i}\right)}{1+\exp\left(\varepsilon_{c}-\alpha H_{i}\right)}-\frac{\exp\left(\varepsilon_{c-1}-\alpha H_{i}\right)}{1+\exp\left(\varepsilon_{c-1}-\alpha H_{i}\right)},for\;c=2,\ldots \ldots ,C-1$$ (10)

$$p\left(\gamma =\frac{C}{H_{i}}\right)=1-\frac{\exp\left(\varepsilon_{c-1}-\alpha H_{i}\right)}{1+\exp\left(\varepsilon_{c-1}-\alpha H_{i}\right)},for\;C$$ (11)

In this study, we examine the marginal effects of predicted probabilities to identify the predictors of club formation. These marginal effects reveal how the probability of affiliation with a convergence club changes when an independent variable alters by one unit, holding all other variables constant. They offer valuable insights into the relationship between dependent and explanatory variables. Past research indicates that results from multinomial logistic regression may not fully elucidate the strength of associations between variables [53].

### 3.4 Data

This study uses annual data from the World Development Indicators (WDI), the World Governance Indicators (WGI), and the World Health Organization for the period 2000 to 2019. Health outcome indicators were selected on peculiar features of African countries and the availability of data. Infant and under-five mortality rate data were selected given the high mortality rates recorded in Africa from 2000 to date. Life expectancy at birth was selected based on the fact that Africa has the lowest life expectancy at birth rate compared to other region worldwide, with significant cross-country variations [54]. The explanatory variables were selected due to their stringent peculiarities in Africa.

The study uses GDP per capita to account for the level of development in countries. Africa is marked by rapid urbanization. The study uses the share of urban population in total population to capture the impact of urbanization on club creation. To account for service coverage, we use the percentage of people using at least basic sanitation services in total population. We also use the proportion of trade in GDP to account for integration. Additionally, we include the percentage of individuals using the internet relative to the total population in the model to evaluate the influence of internet usage on club formation. Variables such as HIV incidence, TB incidence, and mortality associated to Non-communicable diseases were incorporated in the life expectancy model to account for the effects of communicable and non-communicable diseases (CD and NCD) on club creation. Population structure (including the share of the population above 65 and below 15 years old in total population) capture the demographic situation in the region. Studies have shown that Africa is the youngest worldwide, whereas its population aging is increasingly becoming a concern in the region. External health expenditure per capita, in constant (2020) international $ (PPP) was obtained from the World Health Organization database.

We use the principal component analysis (PCA) on six governance indicators to construct the governance quality index. These include regulatory quality, rule of law, government effectiveness, political stability and absence of violence/terrorism, voice and accountability and control of corruption. The domestic general government health expenditure as a share of general government expenditure captures the level of prioritisation of the health sector by the government. It is widely referred to as the Abuja policy instrument.

We expect a positive impact of GDP per capita, sanitation, trade, external health expenditure per capita, and internet usage on club formation such that an increased in these variables is likely to lead to convergence in health outcomes by reducing life expectancy at birth and infant and under-five mortality rate disparities across countries over time [17, 45]. Similarly, we also expect a positive effect of government health expenditure and governance index on club creation [22, 48]. However, it is expected that increasing urbanization, TB incidence, HIV incidence, Mortality due to NCD, younger and older populations negative affect club formation in health outcome [17, 45]. The description, measurement, and sources of the explanatory variables are reported in Panel A of the Appendix 2 in S1 Appendix, while the list of countries is found in Panel B of the Appendix 2 in S1 Appendix.

## 4. Results and discussion

### 4.1 Correlation matrix and principal component analysis results

**Fig 1:**
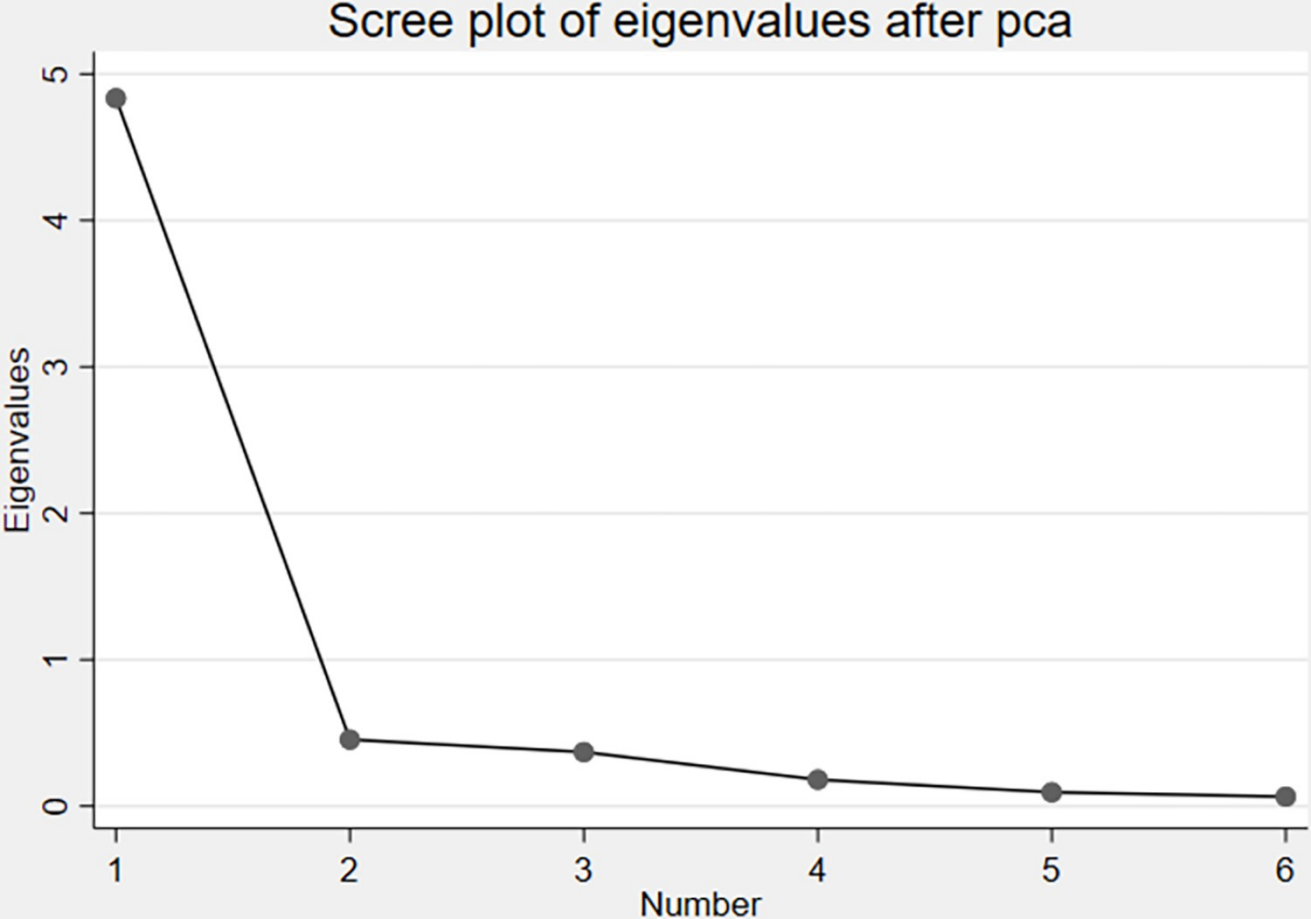
Egenvalues after principal component analysis. Source; Authors’ calculation using data from WGI.

We construct the governance quality index using the PCA method. This method allows us to analyze datasets containing multicollinearity and missing values. It reduces noise in the data and the number of variables in the study by producing independent and uncorrelated data features. It also allows for inspecting clustering algorithms [55]. We first apply the correlation matrix test to see whether the variables are correlated. Table 1 shows the correlation results for the selected governance indicators. The results reveal the presence of high and moderate collinearity among the six indicators, ranging from 0.60 to 0.90. The eigenvalues after PCA are also illustrated in Fig 1. The figure reveals that component 1 (Number 1) has the highest eigenvalue, closer to 5.

**Table 1:** Correlation matrix of governance indicators.

	GEFF	PSAV	CORR	REQ	RUL	VAC
GEFF	1.000					
PSAV	0.639	1.000				
CORR	0.847	0.677	1.000			
REQ	0.903	0.672	0.823	1.000		
RUL	0.904	0.754	0.886	0.885	1.000	
VAC	0.682	0.598	0.709	0.727	0.783	1.000

**Source:** Author’s computation.

Given the correlation results, we employed PCA to construct the governance index. The index allows us to investigate the impact of governance on club formation for the health outcome variables across African countries. The governance indicator PCA results reported in Table 2 suggest that component 1 is preferable compared to the other components because its eigenvalue (4.83) is greater than the eigenvector associated with variables whose loading exceeded 0.40 in absolute value [55].

**Table 2:** Principal component analysis of governance indicators.

**Eigenvalue results**							
**Components**	**Eigenvalue**	**Difference**	**Proportion**	**Cumulative**			
Component 1	4.834	4.380	0.806	0.806			
Component 2	0.455	0.085	0.076	0.882			
Component 3	0.370	0.188	0.062	0.943			
Component 4	0.181	0.086	0.030	0.973			
Component 5	0.095	0.030	0.016	0.989			
Component 6	0.065		0.011	1.000			
**Eigenvector results**							
**Variables**	**Component 1**	**Component 2**	**Component 3**	**Component 4**	**Component 5**	**Component 6**	**Unexplained**
GEFF	0.423	-0.287	-0.321	0.244	-0.575	0.495	0
PVS	0.359	0.897	-0.125	0.148	0.080	0.151	0
CORR	0.419	-0.100	-0.175	-0.841	0.231	0.153	0
REQ	0.422	-0.318	-0.149	0.459	0.696	-0.060	0
RUL	0.442	-0.013	-0.059	-0.012	-0.350	-0.824	0
VAC	0.378	-0.050	0.908	0.020	-0.056	0.163	0

**Source:** Author’s computation using WGI data.

### 4.2 Descriptive statistics

Table 3 presents the mean values of all variables. LEB averaged around 59 years between 2000 and 2019, with a maximum of nearly 77 years, reflecting a relatively lower figure compared to other regions worldwide. The average U5MR was approximately 90 deaths per 1,000 live births, with a range from about 15 to 230 deaths per 1,000 live births, underscoring a persistent challenge in meeting the SDG target of 25 deaths per 1,000 live births [4]. Additionally, IMR averaged about 58 deaths per 1,000 live births, with a maximum of 138.100, further highlighting the significant gap from the SGD target of 25 deaths per 1,000 live births.

**Table 3:** Descriptive statistics of variables of the full sample.

Variables		Abbreviated names	Mean	Std. Dev.	Min	Max
	**Dependent variables**					
Under-five mortality rate		U5MR	90.421	43.835	14.500	224.900
Infant mortality rate		IMR	58.376	24.767	12.500	138.100
Life expectancy at birth		LEB	58.852	7.709	39.441	76.880
	**Independent variables**					
Real GDP per capita		RGDPpc	5316.538	5767.766	715.454	41249.490
Population above 65		POP_G65	3.378	1.398	1.871	11.999
Population below 15		POP_L15	41.267	6.488	17.260	50.264
Urban population		URB	42.218	16.900	8.246	89.741
External health expenditure		EXTHE	27.091	34.054	0.124	223.979
Trade		TRD	66.361	28.426	1.219	175.798
Governance quality index		INS	4.493	2.199	0.000	10.235
Internet usage		NET	11.039	15.177	0.006	84.120
TB incidence		TB	281.710	274.368	11.000	1590.000
HIV incidence		HIV	2.212	3.490	0.010	21.680
Mortality due to non-communicable diseases		NCD	24.969	5.582	13.900	47.900
Government health expenditure		GHE	7.079	3.490	0.633	18.287
Sanitation		STA	36.508	23.792	4.192	96.377

**Note**: The second to third rows show the descriptive statistics of the dependent variables, while the other rows present the descriptive results of the variables used in the marginal effects of the multinomial logistic regression. The first column presents the variables of interests with their abbreviations in parenthesis. The remaining of the columns presents the mean values, the standard deviation, minimum and maximum of each variable, respectively. **Source**: Authors’ computation from WGI, WDI, and WHO data (World Bank).

The average RGDP per capita was about US$5316.54, with a range spanning from US$715.45 to US$41249.49. The considerable standard deviation of about US$5767.77 indicates notable disparities among countries. External health expenditure averaged around US$27.09 per capita. Notably, approximately 41.27 percent of the population was below 15 years old, while about 3.38 percent was 65 years and above, aligning with previous studies indicating Africa as the youngest region globally, posing significant health challenges for its nations [56]. An average of 42.22 percent of the population lived in urban areas, reaching a maximum of about 89.74 percent, indicative of the continent’s rapid urbanization.

Basic sanitation services were used by approximately 36.51 percent of the population while internet usage stood at only 11.04 percent of the population. Concerning infectious and non-infectious diseases, the average HIV incidence was about 2.21 per 1,000 uninfected people while TB incidence averaged 281.71 per 100,000 people. Alarmingly, 24.97 percent of the population aged between 30 and 70 years succumbed to non-communicable diseases between 2000 and 2019. Trade represents approximately 66.36 percent of the GDP in the region. The average quality of governance index across Africa stood at approximately 4.49. Moreover, domestic general government health expenditure accounted for only about 7.08 percent of general government expenditure, significantly falling short of the Abuja target of allocating a minimum of 15 percent of the national budget to the health sector [57].

### 4.3 Convergence results

Tables 4–6 report the convergence results for the infant mortality rate, under-five mortality rate and life expectancy at birth, respectively. The first rows show the convergence results for the overall sample, while the other rows reveal the club clustering, club merging, and the final club classification results. In Table 4, the t-statistics of infant mortality rate for the full sample is -264.13, which is less than the critical value (-1.65). Therefore, the null hypothesis of overall convergence in infant mortality rate in Africa is rejected at the 5 percent significance level. These results indicate an increase cross-country disparity in infant mortality rate over time. These findings align with a previous study that found no evidence in infant mortality rate among African countries [39]. In contrast, other studies found evidence of convergence in infant mortality rate among OECD countries [7].

**Table 4:** Infant mortality rate convergence results.

Sample	Countries	b ^ *Coeff*	SE	t-stat
Overall (40)	All the countries in the sample	-1.025*	0.004	-264.129
Cub 1 (3)	Central African Republic | Nigeria | Sierra Leone	0.530	0.130	4.069
Club 2 (3)	Benin | Congo, Dem. Rep. | Guinea	0.161	0.072	2.230
Club 3 (5)	Botswana | Cote d’Ivoire | Equatorial Guinea | Mali| Mauritania	0.055	0.060	0.909
Club 4 (6)	Angola | Burkina Faso | Cameroon | Comoros | Guinea-Bissau | Togo	0.967	0.071	13.586
Club 5 (8)	Burundi | Gambia, The | Madagascar | Namibia | Niger | Sudan | Eswatini | Zambia	0.214	0.048	4.488
Club 6 (5)	Gabon | Ghana | Kenya | Tanzania | Uganda	1.078	0.089	12.109
Club 7 (5)	Algeria | Mauritius | Rwanda | Senegal | South Africa	0.177	0.077	2.302
Club 8 (3)	Cabo Verde | Morocco | Tunisia	1.001	0.167	6.000
Non-convergent gr. (2)	Chad | Congo, Rep.	-1.624*	0.025	-64.995
**Club Merging**				
Club 1+2		-0.610*	0.032	-19.311
Club 2+3		-0.089	0.048	-1.838
Club 3+4		0.134	0.046	2.920
Club 4+5		-0.342*	0.022	-15.493
Club 5+6		-0.191*	0.016	-11.973
Club 6+7		-0.164*	0.052	-3.175
Club 7+8		-0.200*	0.039	-5.199
**Final club classifications**				
Final club 1 (3)	Central African Republic | Nigeria | Sierra Leone	0.530	0.130	4.069
Final club 2 (3)	Benin | Congo, Dem. Rep. | Guinea	0.161	0.072	2.230
Final club 3 (11)	Angola | Botswana | Burkina Faso | Cameroon | Comoros | Cote d’Ivoire | Equatorial Guinea | Guinea-Bissau | Mali | Mauritania | Togo	0.134	0.046	2.920
Final club 4 (8)	Burundi | Gambia, The | Madagascar | Namibia | Niger | Sudan | Eswatini | Zambia	0.214	0.048	4.488
Final club 5 (5)	Gabon | Ghana | Kenya | Tanzania | Uganda	1.078	0.089	12.109
Final club 6 (5)	Algeria | Mauritius | Rwanda | Senegal | South Africa	0.177	0.077	2.302
Final club 7 (5)	Cabo Verde | Morocco | Tunisia	1.001	0.167	6.000
Divergent club (2)	Chad | Congo, Rep.	-1.624*	0.025	-64.995

Note

* indicates rejection of the null hypothesis of convergence and club convergence merging. SE are the standard errors. The first rows show the result of the full panel sample. The other rows show the results of the initial clubs, the club merging results and the final club classification results. **Source**: Authors’ computation using WDI data.

**Table 5:** Under-five mortality rate convergence results.

Sample	Countries	b ^ *Coeff*	SE	t-stat
Overall (40)	All the countries in the sample	-1.004*	0.004	-255.300
Cub 1 (5)	Central African Republic | Chad | Guinea | Nigeria | Sierra Leone	0.222	0.082	2.711
Club 2 (7)	Benin | Burkina Faso | Congo, Dem. Rep. | Cote d’Ivoire | Equatorial Guinea | Mali | Mauritania	0.008	0.015	0.545
Club 3 (7)	Angola | Cameroon | Comoros | Guinea-Bissau | Niger | Sudan | Togo	0.642	0.082	7.826
Club 4 (9)	Botswana | Burundi | Gambia, The | Ghana | Madagascar | Namibia |	0.348	0.086	4.036
	Eswatini | Tanzania | Zambia			
Club 5 (6)	Congo, Rep. | Gabon | Kenya | Rwanda | Senegal | Uganda	0.393	0.220	1.791
Club 6 (6)	Algeria | Cabo Verde | Mauritius | Morocco | South Africa | Tunisia	0.164	0.079	2.071
**Club Merging**				
Club 1+2		-0.935*	0.014	-65.508
Club 2+3		-0.221*	0.021	-10.553
Club 3+4		-0.480*	0.026	-18.484
Club 4+5		-0.323*	0.050	-6.491
Club 5+6		-0.413*	0.028	-14.973
**Final club classifications**				
Final club 1 (5)	Central African Republic | Chad | Guinea | Nigeria | Sierra Leone	0.2224	0.082	2.711
Final club 2 (7)	Benin | Burkina Faso | Congo, Dem. Rep. | Cote d’Ivoire | Equatorial Guinea | Mali | Mauritania	0.008	0.015	0.545
Final club 3 (7)	Angola | Cameroon | Comoros | Guinea-Bissau | Niger | Sudan | Togo	0.642	0.082	7.826
Final club 4 (9)	Botswana | Burundi | Gambia, The | Ghana | Madagascar | Namibia |	0.348	0.086	4.036
	Eswatini | Tanzania | Zambia			
Final club 5 (6)	Congo, Rep. | Gabon | Kenya | Rwanda | Senegal | Uganda	0.393	0.220	1.791
Final club 6 (6)	Algeria | Cabo Verde | Mauritius | Morocco | South Africa | Tunisia	0.164	0.079	2.071

Note

* indicates rejection of the null hypothesis of convergence and club convergence merging. SE are the standard errors. The first rows show the result of the full panel sample. The other rows show the results of the initial clubs, the club merging results and the final club classification results. **Source**: Authors’ computation using WDI data.

**Table 6:** Life expectancy at birth convergence results.

Sample	Countries	b ^ *Coeff*	SE	t-stat
Overall (40)	All the countries in the sample	-0.108*	0.034	-3.165
Cub 1 (19)	Algeria | Angola | Botswana | Burundi | Congo, Rep. | Gabon | Kenya | Morocco | Namibia | Niger | Rwanda | Senegal | Sierra Leone | South Africa | Eswatini | Tanzania | Tunisia | Uganda | Zambia	0.321	0.068	4.690
Club 2 (8)	Burkina Faso | Cabo Verde | Congo, Dem. Rep. | Guinea | Madagascar | Mali | Mauritius | Sudan	0.098	0.048	2.049
Club 3 (13)	Benin | Cameroon | Central African Republic | Chad | Comoros | Cote d’Ivoire | Equatorial Guinea | Gambia, The | Ghana | Guinea-Bissau | Mauritania | Nigeria | Togo	-0.050	0.054	-0.913
**Club Merging**				
Club 1+2		0.293	0.061	4.852
Club 2+3		-0.153*	0.043	-3.557
**Final club classifications**				
Final club 1 (27)	Algeria | Angola | Botswana | Burkina Faso | Burundi | Cabo Verde | Congo, Dem. Rep. | Congo, Rep. | Gabon | Guinea | Kenya | Madagascar | Mali | Mauritius | Morocco | Namibia | Niger | Rwanda | Senegal | Sierra Leone | South Africa | Sudan | Eswatini | Tanzania | Tunisia | Uganda | Zambia	0.293	0.061	4.852
Final club 2 (13)	Benin | Cameroon | Central African Republic | Chad | Comoros | Cote d’Ivoire | Equatorial Guinea | Gambia, The | Ghana | Guinea-Bissau | Mauritania | Nigeria | Togo	-0.050	0.054	-0.913

Note

* indicates rejection of the null hypothesis of convergence and club convergence merging. SE are the standard errors. The first rows show the result of the full panel sample. The other rows show the results of the initial clubs, the club merging results and the final club classification results. **Source**: Authors’ computation using WDI data.

The clustering test results reveal the existence of eight initial clubs for infant mortality rate, with one divergence club made of Chad and Congo. Some studies reported that Chad’s health indicators are relatively worse than other countries in Africa due to various challenges including civil conflict, healthcare shortages, and lack of maternal and child health. Despite the decline over recent years, infant mortality rate remains extremely high in the country [58]. Additionally, high levels of preventable diseases like malaria, pneumonia, and diarrhoea, as well as bad governance and conflicts in the eastern part of Congo remain significant challenges affecting infant mortality rates in Congo [59]. To avoid overestimating the clubs, the club merging algorithm was performed on the initial convergence clubs. The results show that the number of initial clubs is reduced to seven convergence clubs. This is due to the fact that the merging result of the initial convergence clubs 3 and 4 for the infant mortality rate model was positive and statistically significant, given that t-statistics = 2.920 is greater than the critical value (-1.65). Additionally, the findings in Table 4 also reveal that the distribution of final convergence club exhibits patterns of regional integration and membership. The results suggest that in final Club 1 consisting of three countries, three were members of SEN-SAD, two countries were affiliated to ECOWAS, and one country was a member of EAC. These results are due to overlap of membership among RECs. In the final Club 2, consisting of three members, two are members of SEN-SAD and two from ECOWAS, whereas one is from SADC, and one from ECCAS. In the third final convergence club, six countries were associated with the SEN-SAD region, five with the ECOWAS region, three countries were members of ECCAS, and only two from SADC. In the final convergence Club 4, there were four SADC countries, five COMESA countries, three SEN-SAD countries, and two ECOWAS countries. Half of the selected EAC countries belonged to the final Club 5. Convergence among sub-groups of countries belonging to the same regional grouping was less pronounced in the final clubs 6, and 7, with averages infant mortality rates far below the global target of 25 deaths per 1,000 live births. The divergence club was made of two ECCAS countries and one SEN-SAD country.

Table 5 reports the under-five mortality rate convergence results. The t-statistics of -255.30 indicate that the hypothesis of convergence is not supported. This implies that disparities in under-five mortality rate among African countries widened between 2000 and 2019. These findings are inconsistent previous studies that found evidence of sigma convergence in the under-five mortality rate across 46 SSA between 1990 and 2011 [60]. The clustering test results show the presence of six initial clubs, while the club merging results performed on the initial convergence club show no possibility of forming a bigger convergence club by merging the initial clubs.

The results reveal that the final Club 1 was made of five SEN-SAD countries, three ECOWAS countries, and two ECCAS countries. Similarly, the members of the final Club 2 were predominantly countries from the ECOWAS and SEN-SAD regions, with four members each, and two members of ECCAS. The same pattern was observed in the final Club 3, with five SEN-SAD countries, three ECOWAS countries, and two ECCAS countries. There were also two members form COMESA. The final Club 4 was primarily made of SADC members (six), followed by COMESA members (four), whereas, EAC, ECOWAS, and SEN-SAD had two members each. ECCAS only had one member. The final Club 5 was composed of three COMESA countries, three from EAC, as well as three from ECOWAS. SEN-SAD and IGAD had two members each. In the final Club 6, UMA countries were predominant, with three members, followed by SADC (two countries).

Furthermore, Table 6 also reveals that the null hypothesis of convergence for the overall sample for life expectancy at birth is rejected because the t-statistics of -3.17 is below the critical value (-1.65). Some studies also found full sample divergence in life expectancy and crude mortality rate among OECD countries [18]. However, other studies found evidence of sigma convergence in life expectancy among 46 Sub-Saharan countries [61]. The club clustering results show the existence of three initial clubs made of 19, eight, and 13 countries. The club merging result of the initial convergence clubs 1 and 3 was statistically significant, as shown by the t-statistics = 4.852 > -1.65. These results imply that these initial convergence clubs could merge into larger convergence clubs. Hence, we obtained two final convergence club. Additionally, the final club classification results show that all the selected six EAC countries belong to the final Club 1, whereas almost all the selected SADC and COMESA countries were in the final Club 1, with ten members each. The SEN-SAD region also had ten members in the club, followed by ECOWAS (six members), ECCAS (six members). UMA and the IGAD had each three members. However, among the countries converging to the final Club 2, nine countries were affiliated to SEN-SAD, seven to ECOWAS, and four to ECCAS. The COMESA region only had one member in the club.

#### Relative transition paths for all the clubs

**Fig 2:**
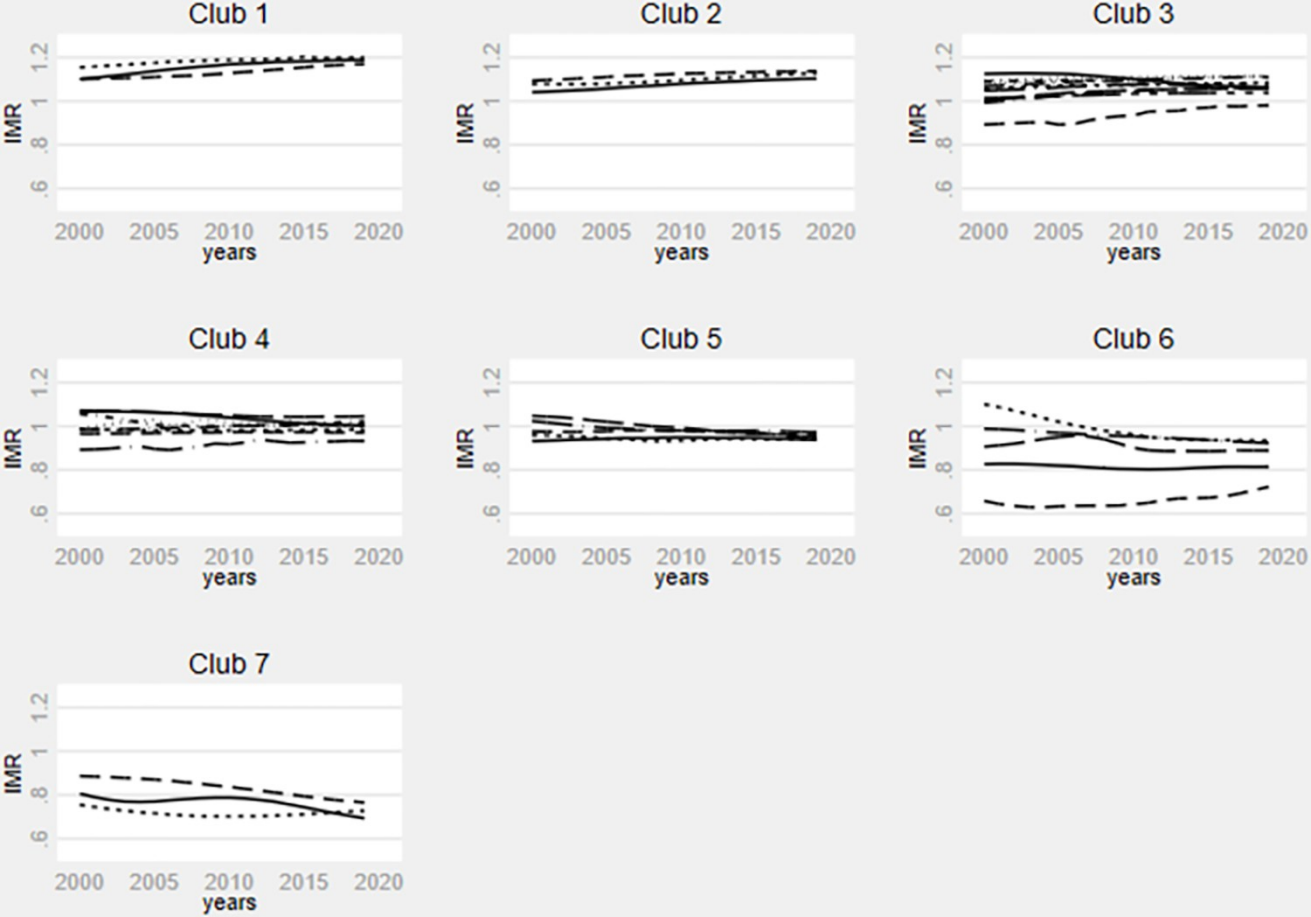
Infant mortality rate relative transition paths by clubs. **Source**: Authors’ calculation using data from WDI.

**Fig 3:**
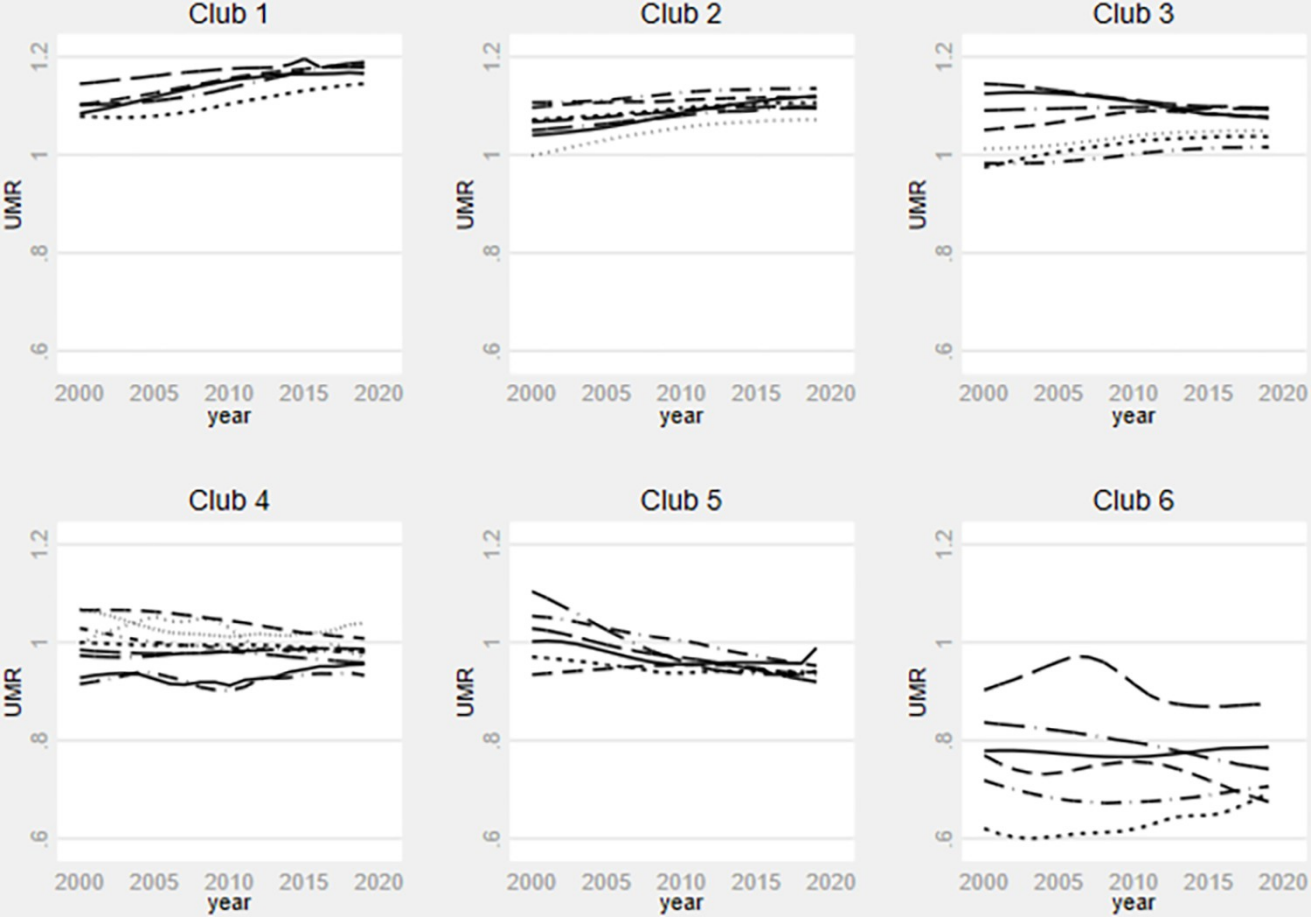
Under-five mortality rate relative transition paths by club. **Source**: Authors’ calculation using data from WDI.

**Fig 4:**
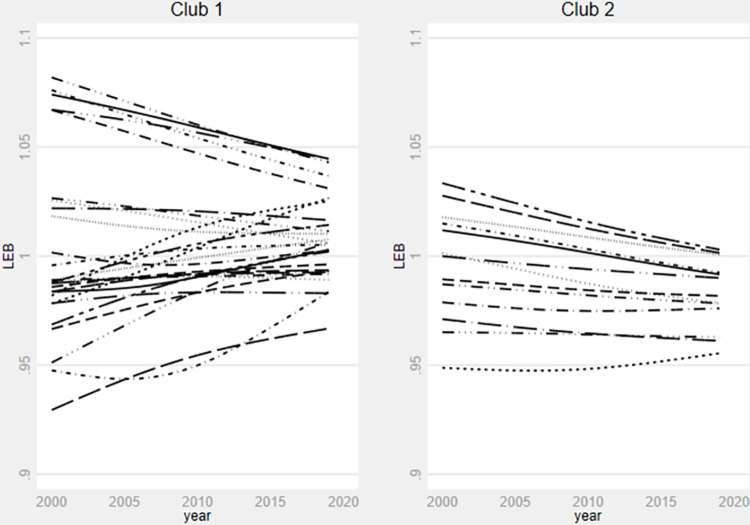
Life expectancy at birth relative transition paths for by clubs. **Source**: Authors’ calculation using data from WDI.

The relative transition paths between the final clubs for infant and under-five mortality rates, as well as life expectancy at birth depict the transitional behaviour of the selected countries, illustrated in Figs 2–4, respectively. The y-axis shows the relative transition parameters while the x-axis denotes the years. In Fig 2, the transition paths of countries within each final club reveal a noticeable catching-up trend over time in the infant mortality rate model. Notably, the year 2015 emerges as a pivotal point. While there is a discernible overall convergence trend in the final clubs post-2015, final clubs 3, 6, and 7 exhibit relatively weaker convergence tendencies, particularly, final club 6 experiences significant volatility among its member countries.

Similarly, Fig 3 shows a catching-up trend among countries within the final clubs for the under-five mortality rate model. Notably, the cross-country disparities seem to significantly diminish from 2015 onwards within each final club, with the exception of the final club 6, where substantial variations persist. Prior to 2015, the influence of existing global, continental, and regional health policies on fostering convergence among countries in final clubs 3, 4, 5, and 6 appears to have been minimal. However, from 2015 onward, health policies appear to exert a significant impact. Fig 4 indicates a reduction in differences in life expectancy at birth over time, particularly from 2015. However, the tendency to converge towards the same steady state within each final club appears relatively weak.

In sum, the transition behaviour of the selected country exhibit reduced volatility post-2015 across the three health outcome variables. These findings are significant because 2015 marked the end of the Millennium Development Goals (MDG) area and the adoption of the SDGs under the 2030 Agenda for Sustainable Development, comprising 169 targets. Unlike the MDGs, the SDGs adopt a more universal approach, emphasizing integration across various actors and domains to achieve global targets. While SDG 3 explicitly addresses health-related issues, over 50 of the 169 targets impact its attainment [62].

Additionally, 2015 also marked the end of the first African Health Strategy (2007–2015). During this pivotal year, the inaugural meeting of the African Union Specialized Technical Committee on Health, Population and Drug Control (STC-HPDC) recommended the development of a revised African Health Strategy for 2016–2030. This updated policy framework (African Health Strategy 2016–2030) is based on several continental and global health commitments and instruments. These include *Agenda 2063: The Africa We Want, the 2030 Agenda for Sustainable Development, various Abuja commitments, calls, and the declarations to fight against AIDS, tuberculosis and malaria in Africa, as well as the Catalytic Framework to End AIDS, TB and Eliminate Malaria in Africa by 2030 [5].*

Furthermore, we measured the means by final clubs to understand the disparities between the clubs. Table 7 reports the results. Regarding infant mortality rate, the mean values of the final club suggest that the gap between most clubs is significant, particularly between final club1 and the rest of the final clubs. On average, countries in final club 7 performed relatively well, with an average of 22.42, below the SDG target of 25 deaths per 1,000 live births. However, final club 1 (composed of the Central African Republic, Nigeria, and Sierra Leone) has the highest average infant mortality rate (98 deaths per 1,000 live births), far from the SDGs’ target. This finding suggests that countries in final club 1 were among the worst performer in terms of infant mortality rate.

**Table 7:** Average of health outcome indicators by final clubs between 2000 and 2019.

	Infant mortality rate			Under-5 mortality rate			Life expectancy at birth		
Clubs	Mean (Sd. Dev.)	Min	Max	Mean (Sd. Dev.)	Min	Max	Mean (Sd. Dev.)	Min	Max
Final club 1	98.528 (15.899)	74.000	138.100	145.100 (27.788)	98.000	224.900	60.259 (8.356)	39.441	76.880
Final club 2	77.843 (12.253)	57.900	106.500	116.506 (25.583)	73.000	187.400	55.931 (5.033)	44.061	64.925
Final club 3	68.946 (17.470)	36.900	121.500	107.782 (36.356)	58.400	224.900			
Final club 4	53.853 (14.722)	30.900	97.000	77.436 (25.552)	41.900	155.900			
Final club 5	47.800 (12.370)	31.500	87.100	73.954 (29.085)	39.700	185.200			
Final club 6	35.420 (19.347)	12.500	109.500	30.112 (16.099)	14.500	79.200			
Final club 7	22.428 (7.678)	12.800	43.900						
Divergence club	66.665 (21.414)	34.700	99.700						
Total	58.376 (24.767)	12.500	138.100	90.421 (43.835)	14.500	224.900	58.852 (7.709)	39.441	76.880

**Note**: Table 7 shows the average values of infant mortality rate, under-five mortality rate and life expectancy at birth by final club. The standard deviations are in parenthesis. The minimum and maximum for each club are also presented. **Source**: Author’s own calculation.

The results also reveal significant differences in under-five mortality rate between the final clubs. Final convergence clubs 1, 2, and 3 had an average mortality rate above 105 deaths per 1,000 live births. Under-five mortality is well pronounced among countries in final club 1. Countries in final club 6 have made significant progress toward achieving the SDGs target, with an average under-five mortality rate of 30.11 deaths per 1,000. However, they still need to put more effort into achieving the target. Finally, the variation in life expectancy at birth across the final clubs is relatively small. On average, countries in final club 1 performed relatively well, averaging 60.26 years. In contrast, the average LEB for final club 2 was about 56 years, which is low.

### 4.4 Marginal effect results

The study used the marginal effects of the ordered logit regression model to investigate the determinants of club formation. The results are presented in Table 8. We report only the marginal effect results because they provide helpful insight into the direction and magnitude of the relationship between the dependent and the explanatory variables. Although the multinomial logit regression helps quantify the relationship between the outcome variable and a set of independent variables, it cannot be used to determine the effects of the independent variables on the variable outcome [63, 64]. The results reported in Table 8 show that the models are significant, with P-values < 0.000. Therefore, the hypothesis that the coefficients of all the variables across the equations are jointly equal to zero is rejected.

**Table 8:** The determinants of club formation.

Variables	Clubs	Infant mortality rate	Under-5 mortality rate	Life expectancy at birth
lRGDPpc	Final club 1	0.025*** (0.005)	0.055*** (0.012)	-0.020 (0.043)
	Final club 2	0.019*** (0.005)	0.038*** (0.009)	0.020 (0.043)
	Final club 3	0.046*** (0.010)	0.008** (0.003)	
	Final club 4	-0.021*** (0.006)	-0.035*** (0.008)	
	Final club 5	-0.040*** (0.008)	-0.039*** (0.009)	
	Final club 6	-0.032*** (0.006)	-0.026*** (0.005)	
	Final club 7	-0.033*** (0.007)		
	Divergent gr.	0.035*** (0.008)		
lPOP_L15	Final club 1	0.129** (0.044)	1.269*** (0.143)	-0.038 (0.240)
	Final club 2	0.097** (0.036)	0.880*** (0.126)	0.038 (0.240)
	Final club 3	0.232** (0.080)	0.176** (0.058)	
	Final club 4	-0.108** (0.040)	-0.808*** (0.113)	
	Final club 5	-0.200** (0.074)	-0.906*** (0.124)	
	Final club 6	-0.160** (0.052)	-0.611*** (0.066)	
	Final club 7	-0.166*** (0.051)		
	Divergent gr.	0.176** (0.060)		
lPOP_G65	Final club 1	0.004 (0.020)	0.306*** (0.062)	0.442*** (0.121)
	Final club 2	0.003 (0.015)	0.212*** (0.048)	-0.442***(0.121)
	Final club 3	0.008 (0.035)	0.043** (0.016)	
	Final club 4	-0.004 (0.016)	-0.195*** (0.043)	
	Final club 5	-0.007 (0.031)	-0.219*** (0.046)	
	Final club 6	-0.005 (0.024)	-0.147*** (0.032)	
	Final club 7	-0.006 (0.025)		
	Divergent gr.	0.006 (0.027)		
lURB	Final club 1	0.011 (0.007)	0.017 (0.016)	-0.614*** (0.056)
	Final club 2	0.009* (0.005)	0.012 (0.011)	0.614*** (0.056)
	Final club 3	0.021* (0.011)	0.002 (0.002)	
	Final club 4	-0.010* (0.005)	-0.011 (0.010)	
	Final club 5	-0.018 (0.010)	-0.012 (0.012)	
	Final club 6	-0.014* (0.008)	-0.008 (0.008)	
	Final club 7	-0.015* (0.008)		
	Divergent gr.	0.016* (0.008)		
lEXTHE	Final club 1	0.014*** (0.003)	0.043*** (0.006)	-0.013 (0.015)
	Final club 2	0.011*** (0.002)	0.030*** (0.004)	0.013 (0.015)
	Final club 3	0.026*** (0.004)	0.006** (0.002)	
	Final club 4	-0.012*** (0.002)	-0.028*** (0.004)	
	Final club 5	-0.022*** (0.003)	-0.031*** (0.004)	
	Final club 6	-0.018*** (0.003)	-0.021*** (0.003)	
	Final club 7	-0.018*** (0.002)		
	Divergent gr.	0.020*** (0.003)		
lTRD	Final club 1	0.017*** (0.003)	-0.001 (0.012)	0.174* (0.099)
	Final club 2	0.013*** (0.003)	-0.001 (0.008)	-0.174* (0.099)
	Final club 3	0.031*** (0.008)	-0.000 (0.001)	
	Final club 4	-0.014*** (0.003)	0.001 (0.008)	
	Final club 5	-0.027*** (0.007)	0.001 (0.008)	
	Final club 6	-0.021*** (0.005)	0.001 (0.006)	
	Final club 7	-0.022*** (0.005)		
	Divergent gr.	0.024*** (0.006)		
lSTA	Final club 1	-0.050*** (0.007)	-0.109*** (0.012)	0.211*** (0.034)
	Final club 2	-0.038*** (0.006)	-0.076*** (0.009)	-0.211*** (0.034)
	Final club 3	-0.090*** (0.009)	-0.015** (0.005)	
	Final club 4	0.042*** (0.007)	0.070*** (0.009)	
	Final club 5	0.078*** (0.007)	0.078*** (0.009)	
	Final club 6	0.062*** (0.008)	0.053*** (0.005)	
	Final club 7	0.064*** (0.007)		
	Divergent gr.	-0.068*** (0.009)		
lNET	Final club 1	-0.003 (0.002)	0.006 (0.006)	0.008 (0.015)
	Final club 2	-0.002 (0.001)	0.004 (0.004)	-0.008 (0.015)
	Final club 3	-0.006 (0.004)	0.001 (0.001)	
	Final club 4	0.003 (0.002)	-0.004 (0.004)	
	Final club 5	0.005 (0.003)	-0.004 (0.004)	
	Final club 6	0.004 (0.003)	-0.003 (0.003)	
	Final club 7	0.004 (0.002)		
	Divergent gr.	-0.004 (0.003)		
lTB	Final club 1			0.275*** (0.027)
	Final club 2			-0.275*** (0.027)
	Final club 3			
	Final club 4			
	Final club 5			
	Final club 6			
	Final club 7			
	Divergent gr.			
lHIV	Final club 1			-0.064*** (0.015)
	Final club 2			0.064*** (0.015)
	Final club 3			
	Final club 4			
	Final club 5			
	Final club 6			
	Final club 7			
	Divergent gr.			
lNCD	Final club 1			0.086 (0.105)
	Final club 2			-0.086 (0.105)
	Final club 3			
	Final club 4			
	Final club 5			
	Final club 6			
	Final club 7			
	Divergent gr.			
lINS	Final club 1	-0.068*** (0.009)	-0.133*** (0.016)	0.186*** (0.047)
	Final club 2	-0.051*** (0.007)	-0.092*** (0.009)	-0.186*** (0.047)
	Final club 3	-0.122*** (0.017)	-0.018** (0.006)	
	Final club 4	0.057*** (0.008)	0.084*** (0.010)	
	Final club 5	0.106*** (0.013)	0.095*** (0.012)	
	Final club 6	0.084*** (0.009)	0.064*** (0.008)	
	Final club 7	0.088*** (0.009)		
	Divergent gr.	-0.093*** (0.013)		
lGHE	Final club 1	-0.030*** (0.006)	-0.079*** (0.014)	0.037 (0.037)
	Final club 2	-0.023*** (0.005)	-0.055*** (0.011)	-0.037 (0.037)
	Final club 3	-0.055*** (0.011)	-0.011** (0.004)	
	Final club 4	0.026*** (0.006)	0.051*** (0.010)	
	Final club 5	0.047*** (0.008)	0.057*** (.010)	
	Final club 6	0.038*** (0.007)	0.038*** (0.007)	
	Final club 7	0.039*** (0.007)		
	Divergent gr.	-0.042*** (0.008)		
**No. of obs.**		**772**	**772**	**772**
**P-Values**		**0.000**	**0.000**	**0.000**

Note

***, **, and * show the statistical significance at 1%, 5%, and 10%, respectively. The first column shows the code of the explanatory variables used; the second column show the final clubs. The third, fourth and fifth columns show the marginal effects results of the infant mortality rate, under-five mortality rate and life expectancy at birth models, respectively. The standard errors are in parenthesis. **Source**: Authors’ own computation

#### Infant mortality rate results

The marginal effects of the explanatory variables show mixed results across the clubs. In the IMR model analysis, factors such as elderly population, urbanization, and internet usage are statistically insignificant in determining the probability of belonging to the final clubs. However, an increase of one unit in initial lRGDPpc leads to a higher likelihood of belonging to final clubs 1, 2, 3 and the divergent club by 0.03, 0.02, 0.005, and 0.04 log points respectively. Conversely, it decreases the probability of belonging to final clubs 4, 5, 6, and 7 by 0.02, 0.17, 0.04, 0.03, and 0.03 log points respectively. Furthermore, the results reveal that a unit increase in initial proportion of the population below 15 years raises the likelihood of membership in final clubs 1, 2, 3, and the divergent club by 0.13, 0.10, 0.23, and 0.18 log points respectively. However, it reduces the probability of affiliation with final clubs 4, 5, 6, and 7 by 0.11, 0.20, 0.16, and 0.17 long points respectively. Previous studies have also highlighted the significance of macroeconomic factors, such as GDP per capita and population structure, as key drivers of health outcomes and health outcome convergence across EU and WAEMU countries [17, 52]. A unit raise in the initial percentage of people with access to basic sanitation decreases the probability of membership in final clubs 1, 2, 3, and the divergence club. Conversely, it increases the likelihood of belonging to final clubs 4, 5, 6, and 7. The marginal effects of the other control variables also exhibit varied outcomes.

Furthermore, the results indicates that a one-unit improvement in lINS decreases the probability of affiliation with final clubs 1, 2, 3, and the divergent club by 0.07, 0.05, 0.12, and 0.09 log points respectively. Conversely it raises the likelihood of membership in final clubs 4, 5, 6, and 7 by 0.06, 0.11, 0.08, and 0.09 log points respectively. Notably, many countries included in final clubs 1, 2, and 3 exhibited comparatively low overall governance scores between 2010 and 2019. These include Nigeria, DRC, Angola, Cameroon, and Comoros, to mention a few (Mo Ibrahim Foundation 2020). These findings align with previous studies that demonstrating the positive impact of governance on health outcome convergence among ECOWAS countries [45].

Additionally, an increase of one unit in initial general government health expenditure as a percentage of general government expenditure significantly decreases the probability of affiliation with final clubs 1, 2, 3, and the divergent club by 0.03, 0.02, 0.06, and 0.04 log points, respectively. However, it raises the likelihood of membership in final club 4, 5, 6, and 7 by 0.03, 0.05, 0.04, and 0.04 log points, respectively. Prior evidence has also demonstrated that increased government health spending is associated with improved health outcomes in Africa, particularly among ECOWAS countries [22]. Moreover, increasing public health spending has been recognized as a vital factor in reducing infant mortality across WAEMU countries [52].

#### Under-five mortality rate results

The findings reveal that variables such as urbanization, trade, and internet usage are not significant in explaining the formation of the final clubs. However, the effects of several control variables align with expectations. For example, a one unit increase in initial lPOP_L15 significantly increases the probability of belonging to final clubs 1, 2, and 3 by 1.27, 0.09, and 0.18 log points, respectively. However, it decreases the likelihood of belonging to final clubs 4, 5, and 6 by 0.81, 0.91, and 0.61 log points, respectively. Additionally, when lPOP_G65 increases by one unit, the probability of membership in final clubs 1, 2, and 3 increases by 0.31, 0.21, and 0.04 log points respectively. But it decreases the likelihood of belonging to final clubs 4, 6, and 6 by 0.20, 0.22, and 0.15 log points, respectively. A similar trend is observed concerning external health expenditure per capita.

Similarly, a one-unit increase in initial RGDPpc raises the probability of belonging to final clubs 1, 2, and 3 by 0.06, 0.04, and 0.008 log point respectively. Conversely, it decreases the likelihood of membership in final clubs 4, 5, and 6 by 0.04, 0.05, and 0.06 log points respectively. However, an increase in access to basic sanitation reduces the likelihood of affiliation with final clubs 1, 2, and3, while raising the probability of belonging to final clubs 4, 5, and 6. A positive impact of GDP per capita on health outcome convergence was also evident among ECOWAS countries [45].

In analysing our variable of interest, the results reveal that a one-unit increase in the governance index leads to a decreased probability of affiliation with final clubs 1, 2, and 3 by 0.13, 0.09, and 0.02 log points, respectively. However, it increases the likelihood of belonging to final clubs 4, 5, and 6 by 0.0.08, 0.10, and 0.06 log points, respectively. Governance has also been shown to positively influence convergence in the ECOWAS region [11]. Furthermore, as government spending on health increases by one unit, there is a lower probability of belonging to final clubs 1, 2, and 3 by 0.0.08, 0.0.06, and 0.01 log points, respectively. However, it raises the likelihood of belonging to final club 4, 5, and 6 by 0.05, 0.06, and 0.04 log points respectively. The literature also supports a positive correlation between government health expenditure and health outcomes [22].

#### Life expectancy at birth results

In the LEB model analysis, the results reveal that real GDP per capita, the proportion of younger population, external health expenditure per capita, mortality associated with non-communicable diseases, and internet usage are insignificant in determining the creation of the final clubs. However, our findings also show that a unit increase in initial proportion of elderly population raises the likelihood of membership in final club 1 by 0.44 log points, while reducing the probability of affiliation with final club 2 by 0.44 log points. Similarly, a one-unit increase in the share of trade in GDP increases the probability of affiliation with final club 1. But it reduces the likelihood of membership to final club 2. A similar pattern is observed concerning the access to basic sanitation services, TB incidence,. These results are consistent with some existing studies that found a positive relationship between urbanization and sanitation and life expectancies [22, 50]. However, a unit increase in initial lURB reduces the probability of belonging to final club 1 by 0.61 log points. Still, it increases the likelihood of membership to final club 2 by 0.61 log points. There are mixed effects regarding the other control variables.

When looking at the variables of interest, government health expenditure as a percentage of general government expenditure results are insignificant for all the clubs. These findings are inconsistent with existing studies that found that government health expenditure is positively associated with health outcome convergence [22]. However, a unit increase in governance index leads to a higher probability of 0.19 log points belonging to final club 1. But there is a lower probability of 0.19 to be affiliated with final club 2. There is existing evidence that governance is positively associated with life expectancies [45]. Similarly, political democratization, economic development, and nutritional improvements have a long-term positive effect on life expectancies [48].

*Robustness check of the estimates*. We used the marginal effects of the multinomial logistic model regression to check the robustness of the ordered logit regression results. The results are reported in Appendix 1 in S1 Appendix. Overall, the results reveal that the signs, magnitude, and significance of the estimated coefficients are different for most cases between the marginal effect of the multinomial logistic regression and the ordered logit model regression, particularly for the infant and under-five mortality rate models. The marginal effects of the ordered logic regression are generally smaller than those of the multinomial logistic regression for both models. This can be explained by the fact that the multinomial regression model does account for the ordered aspect of the final clubs compared to the ordered logic regression model. However, the results from the marginal effects of the multinomial logic regression for the life expectancy model are similar to the ordered logit ones in terms of the significance, signs and magnitudes.

## 5. Result discussion

The convergence analysis across the overall sample reveal evidence of divergence in Africa concerning infant and under-five mortality rates, as well as life expectancy at birth. This implies that African countries are not collectively progressing toward the consistent health outcomes, despite shared policies and interventions at the continental level. Thus, the benefits of health integration predominantly favor countries with comparatively higher levels of development within the continent. The persistent health outcome disparities make the African region a microcosm of global health inequalities. Although the African Union and its partners have made considerable efforts to promote health integration and cooperation, challenges persist in terms of regulations, infrastructure, and policy implementation [5]. Previous reports have also highlighted disparities in health outcomes among African countries [56]. It is crucial to recognize that these persistent gaps in health outcomes incur substantial financial costs and impede progress toward achieving SDGs and UHC. Moreover, they exert a detrimental influence on socio-economic development of Africa [60].

We found evidence of convergence clubs for each variable of interest. There are seven final convergence clubs for infant mortality rate, six for under-five mortality rate, and two for life expectancy at birth. Notably, Nigeria, the Central African Republic, and Sierra Leone belong to final club 1, characterized by the highest infant and under-five mortality rates. These high rates are partly attributed to internal conflict, post-conflicts, and civil unrest, leading to increased mortality rates, particularly among children [4]. Such challenges may hinder countries in final club 1 from catching up with those countries in final clubs with relative lower infant and under-five mortality rates. Furthermore, there is also evidence of intra-club disparities, consistent with studies that have found evidence of cross-country variations in health outcomes [56].

It is worrisome that all sub-groups of countries in the under-five mortality rate model converge towards higher mortality rates among children under five. On average, no final club regarding this model met the global target of 25 deaths per 1,000 births. However, the transition paths of countries in different final clubs for the health outcome variables exhibit reduced volatile post-2015. This is significant as it indicates that efforts by the African Union, the regional economic groupings, as well as initiatives such as SDGs and UHC have played pivotal roles in guiding some countries towards similar health outcomes trajectories.

The results also indicate that the distribution of final clubs exhibit patterns of uniformity among countries within the same regional grouping. In the infant mortality rate model, countries in SEN-SAD and ECOWAS appear to be affiliated to the final club characterized by the highest rates of child mortality. However, countries in SADC and EAC tend to be more prevalent in sub-groups with relatively lower infant mortality rates. Similarly, the convergence analysis for under-five mortality model reveals that countries in SEN-SAD, ECOWAS, and ECCAS regions predominantly affiliate with the final clubs 1, 2, and 3, exhibiting under-five mortality rate exceeding 100 deaths per 1,000 live births.

Regarding life expectancy at birth, all the selected EAC countries belong to final clubs characterized by the highest life expectancy. Additionally, a significant portion of the selected SADC, AMU, IGAD, and COMESA countries are members of the final Club 1. Moreover, there is a notable representation from SEN-SAD, ECOWAS, and ECCAS countries in the final Club 1. Final Club 2 is primarily composed of countries from ECOWAS, SEN-SAD and ECCAS regions. These results suggest that common policies and interventions at the regional level tend to effectively promote convergence among member states. However, disparities in health policies, the level of health prioritization, and the mechanisms they implement also impact the progress toward achieving better health outcomes among member states. The literature indicates a low prioritisation of health in ECOWAS countries, evidenced by high mortality rates [22]. Additionally, we found evidence that countries within the same African regional grouping may also diverge. Hence, the results imply that convergence does not solely occur among countries within the same regional economic community.

The marginal effects derived from ordered logit regression model reveal that increasing proportion of young and elderly population, external health expenditure per capita, and real GDP per capita notably raises the likelihood of membership in the final clubs characterized by poorer child mortality rates. However, an increase in the proportion of people with access to basic sanitation services leads to a increased probability of belonging to final clubs with relatively better health outcomes.

Life expectancy model results reveal that increased population aging, TB incidence, access to basic sanitation services and trade are associated with a higher likelihood of belonging to final clubs with relatively higher life expectancy at birth. However, increased urbanization and HIV incidence lead to increased probability of membership in final clubs with relatively lower life expectancy. These results are consistent with previous studies that found that urbanisation in many countries including Nigeria, Sierra Leone, Cameroon, DR Congo, Guinea-Bissau (mostly belonging to the final convergence clubs with the worse health outcomes) is chaotic, characterised by high rates of poverty, unemployment, and inequality due to the lack of massive social, economic and infrastructural transformations. There are significant health crises resulting from inadequate safe water supply, poor sanitation, and high prevalence of infectious and non-infectious diseases [45, 64].

The quality of governance emerges as a significant determinant of club formation, exerting a positive influence on the probability of converging towards final clubs with comparatively better health outcomes. However, it reduces the likelihood of belonging to final clubs with comparatively poorer health outcomes. Previous studies found a positive relationship between good governance and both health outcomes and health outcome convergence [45, 48]. However, the results indicate a reduced probability of membership in final clubs with worse health outcomes as the governance quality index increases by a unit. This can be attributed to the prevalence of poor governance in many of these countries [65]. In this line, resource allocation to the health sector in settings characterized by poor governance may prove insufficient in improving health outcomes. This inadequacy is primarily attributed to inefficiency and poor healthcare service delivery, mis-allocation of resources, mismanagement, corruption, and deficient health infrastructures [66, 67].

Furthermore, our findings indicate that domestic government health expenditure plays a crucial role in determining club formation. An increase in domestic public spending on health positively influences the probability of belonging to final clubs with relatively better health outcomes, while significantly reducing the likelihood of affiliation with final clubs exhibiting worse health outcomes. These results underscore the importance of the 2001 Abuja Declaration policy in shaping club formation dynamics. Since 2000, approximately nineteen countries have witnessed a decline in their relative government allocation to health. These countries include Benin, Sao Tome and Principe, Botswana, Cameroon, Comoros, Equatorial Guinea, Mauritania, Togo, Niger, Zambia, Chad, Sierra Leone, Central African Republic, Senegal, Tanzania, Cabo Verde, Rwanda, Zimbabwe, and Mozambique [68]. Most of these countries belong to final clubs characterized by the poorest health outcomes. However, countries like Burundi, Gambia, Madagascar, Namibia, and Eswatini, to mention a few, have demonstrated increases in health spending over the years.

The share of government health expenditure to general government spending not only serves as an instrument of the Abuja Declaration but also reflects the level of health priority in government spending. Our results suggest that many countries in the final clubs with worse health outcomes exhibit low political will concerning health financing reforms. In most countries, governments allocate significant resources to the defence sector, partly driven by internal conflict [69]. Most countries affiliated with the final clubs displaying the worst health outcomes faced numerous challenges, including poverty, high unemployment, food insecurity, inequality, and climate change, which pose obstacles to prioritizing health amidst competing demands. In addition, the deteriorating macroeconomic conditions resulting from the COVID-19 pandemic, the effects of the war in Ukraine, and mounting inflation significantly impede these countries’ abilities to raise enough funds for the health sector [70]. These recent deteriorating macroeconomic downturns have exacerbated existing disparities in health outcomes among countries, particularly in the African region [70].

## 6. Conclusion and policy implications

This paper examines the convergence hypothesis in three health outcomes among 40 African countries from 2000 to 2019. The results show the absence of overall convergence in infant mortality rate, under-five mortality rate, and life expectancy at birth, underscoring the persistent disparities in health outcomes across Africa. Despite the implementation of various of common initiatives and policies aimed at improving health outcomes and acheiving health-related SDGs and UHC, these findings reveal enduring discrepancies among African countries. Consequently, African health systems face additional pressure suggesting that progress toward health integration in the continent remains far from realization.

The results reveal the presence of seven final clubs for infant mortality rates, six for under-five mortality rates, and two for life expectancy at birth. However, in the infant mortality model, Chad and Condo exhibit divergence. The evidence of convergence clubs suggests that sub-groups of countries with similar characteristics experience diminishing health outcome inequalities over time. Furthermore, significant disparities between final clubs for the three variables of interest imply that, “catch-up effects” may be more prominent within final club rather than between them. Additionally, our results indicate convergence among countries within the same regional grouping across all three models. Generally, countries in ECOWAS, SEN-SAD, and ECCAS are predominantly associated with final clubs with the worst health outcomes, whereas several countries in the SADC, EAC, and COMESA regions affiliate with final clubs exhibiting relatively better health outcomes. However, we also observe that convergence does not solely occur among countries within the same regional economic community. There are different regional groupings within the final clubs.

The study also used the marginal effects from ordered logit regression to identify the factors influencing the probability of belonging to specific final clubs. The results reveal varied impact across different final clubs. Among the control variables, the findings show that increasing GDP per capita, population structure, external resources on health, and trade positively influence the likelihood of belonging to final clubs with comparatively higher child mortality rates. However, access to basic sanitation influence the probability of affiliation with final clubs with comparatively lower rates. Additionally, increasing proportion of elderly population, TB incidence, access to basic sanitation services, and share of trade in GDP is associated with a higher probability of membership in final club with relatively higher life expectancy at birth, whereas increased urbanization and HIV incidence raises the likelihood of belonging to final clubs with relatively lower life expectancy.

Furthermore, the findings of governance quality suggest that an increase in the governance quality index positively influences a country’s probability of belonging to final clubs with relatively better health outcomes. Conversely, it negatively affects the likelihood of affiliation with final clubs with poorer health outcomes. In addition, our results reveal that the Abuja policy instrument significantly drives convergence for final clubs for the child mortality models. While it positively influences the probability of membership in final convergence clubs with relatively better child mortality rates, adverse effects are observed for final clubs with worse mortality rates.

The objectives addressed in this study and the methods used are relevant in the African context, and the policy implications and recommendations are essential for achieving the AU’s vision of an integrated and prosperous Africa, free of its heavy double burden of infectious and non-infectious diseases and injuries. They are also crucial for the socio-economic development of the continent. In this context, the convergence method used in this study can be used as a critical assessment tool for monitoring the progress of existing health policies and initiatives. This assists in assessing how successful the existing policies are in meeting the health goals at the regional and continental levels. The convergence test suggests that policies undertaken, such as the Abuja Declaration policy have not been successful in promoting convergence at the continental level. However, The policy significantly affects the probability of converging to a particular sub-group. Its effect is positive and significant for to final clubs with better health outcomes. Thus, there is a need for homogeneous health policies for each convergence club, and the focus should be on converging countries that belong to the same regional grouping within the clusters. However, there is also a need for country-specific policies for the diverging countries. The results also suggest that strategies promoting higher health prioritization should be encouraged within each final club. Given the limited fiscal space of many African countries, initiatives and policies prioritizing the health sector in governments’ budget allocation and reallocation need to be considered. In addition, a monitoring mechanism should be adopted, particularly at the regional level to track the progress of member states regarding initiatives undertaken to ensure that countries meet their health commitments.

Moreover, countries in final convergence clubs with worse health outcomes should develop urbanization plans that promote productivity and adequate living standard in cities. Such initiatives should focus on planned and regulated urbanization. Access to high-quality service coverage is critical to improving health outcomes. Therefore, policies that strive to improve access to access to basic sanitation should also be encouraged in the different final clubs. This is critical for reducing sanitation-related diseases that can worsen health outcomes and health outcomes disparities. Given the significant impact of the governance quality index on health outcome convergence, policies that promote good governance should be encouraged. These include anti-corruption policies that aim to prevent, detect, and prosecute corruption. Governments should also strive to improve their effectiveness. Greater participation and accountability should also be encouraged.

However, this study has some limitations. The study did not control for important variables, including education and climate change which may also affect the probability of belonging to particular final clubs. The study did not also investigate the convergence hypothesis regarding other essential health outcome indicators such as mortality between 1 and 4, life expectancy at 5, and healthy life expectancy. However, the health outcome indicators used in this study are still widely used to examine health outcomes within and between countries. Although our study attempted to fill the gap in the literature on health outcome convergence, we believe that the current paper can be improved by addressing the issues mentioned above in future research.

## Supporting information

S1 Appendix (DOCX)
