# Supplementary material for: Health outcome convergence and the roles of public health financing and governance in Africa
Source: PLoS One. 2024 Oct 15;19(10):e0312089. doi: 10.1371/journal.pone.0312089 (PMC11478915; doi:10.1371/journal.pone.0312089)
Supplement: S1 Appendix — (DOCX) [file pone.0312089.s001.docx]

# Appendices

# Appendix 1: Marginal effect derived from multinomial logit regression model

| **Variables** | **Clubs** | **Infant mortality rate** | **Under-5 mortality rate** | **Life expectancy at birth** |
| --- | --- | --- | --- | --- |
| lRGDPpc | Final club 1 | -0.067***  (0.017) | 0.005  (0.026) | -0.020  (0.043) |
|  | Final club 2 | -0.061**  (0.024) | 0.032  (0.030) | 0.020  (0.043) |
|  | Final club 3 | -0.060  (0.038) | -0.008  (0.027) |  |
|  | Final club 4 | -0.097**  (0.032) | -0.101***  (0.031) |  |
|  | Final club 5 | 0.280***  (0.052) | 0.072*  (0.040) |  |
|  | Final club 6 | -0.000**  (0.000) | -0.000***  (0.000) |  |
|  | Final club 7 | -0.000**  (0.000) |  |  |
|  | Divergence club | 0.005  (0.011) |  |  |
| lPOP_L15 | Final club 1 | -0.429**  (0.206) | 1.010***  (0.224) | -0.038  (0.240) |
|  | Final club 2 | 1.377***  (0.145) | 1.206***  (0.309) | 0.038  (0.240) |
|  | Final club 3 | -1.979***  (0.334) | -0.080  (0.267) |  |
|  | Final club 4 | 1.136***  (0.204) | -1.216***  (0.244) |  |
|  | Final club 5 | -0.368  (0.270) | -0.920**  (0.349) |  |
|  | Final club 6 | -0.000**  (0.000) | -0.000***  (0.000) |  |
|  | Final club 7 | -0.000**  (0.000) |  |  |
|  | Divergence club | 0.263  (0.160) |  |  |
| lPOP_G65 | Final club 1 | 0.098  (0.076) | 0.658***  (0.083) | 0.442***  (0.121) |
|  | Final club 2 | 0.551***  (0.061) | 0.556***  (0.118) | -0.442***  (0.121) |
|  | Final club 3 | -0.411***  (0.101) | -0.400***  (0.099) |  |
|  | Final club 4 | 0.449***  (0.076) | -0.388***  (0.076) |  |
|  | Final club 5 | -0.533***  (0.078) | -0.425**  (0.137) |  |
|  | Final club 6 | 0.000**  (0.000) | 0.000***  (0.000) |  |
|  | Final club 7 | 0.000**  (0.000) |  |  |
|  | Divergence club | -0.153**  (0.049) |  |  |
| lURB | Final club 1 | 0.091***  (0.027) | -0.047  (0.033) | -0.614***  (0.056) |
|  | Final club 2 | 0.114**  (0.045) | 0.003  (0.037) | 0.614***  (0.056) |
|  | Final club 3 | 0.065  (0.049) | 0.106**  (0.051) |  |
|  | Final club 4 | -0.111***  (0.033) | 0.107**  (0.038) |  |
|  | Final club 5 | -0.175***  (0.038) | -0.170***  (0.039) |  |
|  | Final club 6 | -0.000***  (0.000) | -0.000**  (0.000) |  |
|  | Final club 7 | 0.000**  (0.000) |  |  |
|  | Divergence club | 0.015  (0.030) |  |  |
| lEXTHE | Final club 1 | 0.030**  (0.009) | 0.043**  (0.016) | -0.013  (0.015) |
|  | Final club 2 | -0.010  (0.009) | 0.057***  (0.014) | 0.013  (0.015) |
|  | Final club 3 | -0.013  (0.016) | -0.132***  (0.016) |  |
|  | Final club 4 | 0.047***  (0.015) | 0.097***  (0.014) |  |
|  | Final club 5 | -0.058***  (0.012) | -0.065***  (0.013) |  |
|  | Final club 6 | -0.000**  (0.000) | -0.000***  (0.000) |  |
|  | Final club 7 | -0.000  (0.000) |  |  |
|  | Divergence club | 0.004  (0.006) |  |  |
| lTRD | Final club 1 | -0.045**  (0.021) | -0.016  (0.026) | 0.174*  (0.099) |
|  | Final club 2 | 0.023  (0.024) | 0.045  (0.037) | -0.174*  (0.099) |
|  | Final club 3 | 0.092**  (0.036) | -0.179***  (0.025) |  |
|  | Final club 4 | -0.024  (0.027) | 0.131***  (0.034) |  |
|  | Final club 5 | -.0108***  (0.020) | 0.019  (0.044) |  |
|  | Final club 6 | -0.000**  (0.000) | -0.000*  (0.000) |  |
|  | Final club 7 | 0.000**  (0.000) |  |  |
|  | Divergence club | 0.061***  (0.015) |  |  |
| lSTA | Final club 1 | 0.031**  (0.012) | -0.083***  (0.016) | 0.211***  (0.034) |
|  | Final club 2 | -0.105***  (0.013) | 0.002  (0.022) | -0.211***  (0.034) |
|  | Final club 3 | 0.172***  (0.033) | -0.132***  (0.023) |  |
|  | Final club 4 | 0.157***  (0.024) | 0.091***  (0.025) |  |
|  | Final club 5 | -0.181***  (0.029) | 0.122***  (0.024) |  |
|  | Final club 6 | 0.000**  (0.000) | -0.000**  (0.000) |  |
|  | Final club 7 | -0.000  (0.000) |  |  |
|  | Divergence club | -0.074***  (0.019) |  |  |
| lNET | Final club 1 | -0.011  (0.010) | 0.018*  (0.010) | 0.008  (0.105) |
|  | Final club 2 | 0.021**  (0.008) | -0.001  (0.010) | -0.008  (0.105) |
|  | Final club 3 | -0.057***  (0.012) | 0.042***  (0.008) |  |
|  | Final club 4 | 0.022**  (0.011) | -0.049***  (0.010) |  |
|  | Final club 5 | 0.021**  (0.008) | -0.010  (0.012) |  |
|  | Final club 6 | 0.000***  (0.000) | -0.000**  (0.000) |  |
|  | Final club 7 | 0.000  (0.000) |  |  |
|  | Divergence club | 0.003  (0.002) |  |  |
| lTB | Final club 1 |  |  | 0.275***  (0.027) |
|  | Final club 2 |  |  | -0.275***  (0.027) |
|  | Final club 3 |  |  |  |
|  | Final club 4 |  |  |  |
|  | Final club 5 |  |  |  |
|  | Final club 6 |  |  |  |
|  | Final club 7 |  |  |  |
|  | Divergence club |  |  |  |
| lHIV | Final club 1 |  |  | -0.064***  (0.015) |
|  | Final club 2 |  |  | 0.064***  (0.015) |
|  | Final club 3 |  |  |  |
|  | Final club 4 |  |  |  |
|  | Final club 5 |  |  |  |
|  | Final club 6 |  |  |  |
|  | Final club 7 |  |  |  |
|  | Divergence club |  |  |  |
| lNCD | Final club 1 |  |  | 0.086  (0.105) |
|  | Final club 2 |  |  | 0.086  (0.105) |
|  | Final club 3 |  |  |  |
|  | Final club 4 |  |  |  |
|  | Final club 5 |  |  |  |
|  | Final club 6 |  |  |  |
|  | Final club 7 |  |  |  |
|  | Divergence club |  |  |  |
| lINS | Final club 1 | -0.093***  (0.021) | -0.162***  (0.021) | 0.186***  (0.047) |
|  | Final club 2 | -0.007  (0.017) | -0.087**  (0.033) | -0.186***  (0.047) |
|  | Final club 3 | -0.044  (0.044) | -0.103***  (0.028) |  |
|  | Final club 4 | 0.007  (0.039) | 0.085**  (0.035) |  |
|  | Final club 5 | 0.175***  (0.031) | 0.267***  (0.038) |  |
|  | Final club 6 | 0.000**  (0.000) | 0.000***  (0.000) |  |
|  | Final club 7 | 0.000*  (0.000) |  |  |
|  | Divergence club | -0.039***  (0.011) |  |  |
| lGHE | Final club 1 | -0.004  (0.013) | -0.018  (0.020) | 0.037  (0.037) |
|  | Final club 2 | -0.101***  (0.012) | -0.142***  (0.024) | 0.037  (0.037) |
|  | Final club 3 | -0.178***  (0.027) | -0.046*  (0.024) |  |
|  | Final club 4 | 0.273***  (0.028) | 0.293***  (0.029) |  |
|  | Final club 5 | 0.025  (0.018) | -0.088**  (0.030) |  |
|  | Final club 6 | -0.000**  ().000) | -0.000***  (0.000) |  |
|  | Final club 7 | -0.000**  (0.000) |  |  |
|  | Divergence club | -0.014*  (0.009) |  |  |
| **No. of obs.** |  | **772** | **772** | **772** |
| **Prob > chi2** |  | **0.000** | **0.000** | **0.000** |

**Note**: ***, **, and * show statistical significance at 1%, 5%, and 10%, respectively. The first column show the code of the explanatory variables used, the second column show the final clubs. The third, fourth and fifth columns show the marginal effects results of the ordered logit model for the infant mortality rate, under-five mortality rate and life expectancy at birth models, respectively. The standard errors are in parenthesis. **Source**: Authors’ own computation

# Appendix 2: List of variables and countries

|  | **Panel A: List of variables and description** | | |
| --- | --- | --- | --- |
| **Variable names** | **Abbreviation** | **Description** | **Sources** |
|  |  | **Health expenditure variables** |  |
| Under-five mortality rate | U5MR | Mortality rate, under-5 (per 1,000 live births) | WDI database |
| Infant mortality rate | IMR | Mortality rate, infant (per 1,000 live births) | WDI database |
| Life expectancy at birth | LEB | Life expectancy at birth, total (years) | WDI database |
|  |  | **Explanatory variables** |  |
| Urban population | URB | Urban population (% of total population) | WDI database |
| Population below 15 | POP_L15 | Population ages 0-14 (% of total population) | WDI database |
| Population above 65 | POP_G65 | Population ages 65 and above (% of total population) | WDI database |
| Regulatory quality | REQ | Regulatory Quality | WGI database |
| Rule of law | RUL | Rule of Law | WGI database |
| Government effectiveness | GEFF | Government Effectiveness | WGI database |
| Political stability | PSAV | Political Stability and Absence of Violence/Terrorism | WGI database |
| Control of corruption | CORR | Control of Corruption | WGI database |
| Voice and accountability | VAC | Voice and Accountability | WGI database |
| External health expenditure | EXTHE | External health expenditure as a percentage of GDP | WHO database |
| Real GDP per capita | RGDpc | GDP per capita, PPP (constant 2017 international $) | WDI database |
| Trade | TRD | Trade (% of GDP) | WDI database |
| Sanitation | STA | People using at least basic sanitation service (% of population) | WDI database |
| Government health expenditure | GHE | Domestic general government health expenditure (% of general government expenditure) | WDI database |
| Internet usage | NET | Individual using the internet (% of population) | WDI database |
| Non-communicable diseases | NCD | Mortality from CVD, cancer, diabetes, CRD between exact ages 30 and 70 (%) | WDI database |
| HIV incidence | HIV | Incidence of HIV, all (per 1,000 uninfected people) | WDI database |
| TB incidence | TB | Incidence of tuberculosis (per 100,000 people) | WDI database |

**Panel B: List countries**

| Algeria | Guinea | Togo |
| --- | --- | --- |
| Angola | Guinea-Bissau | Tunisia |
| Benin | Kenya | Uganda |
| Botswana | Madagascar | Zambia |
| Burkina Faso | Mali |  |
| Burundi | Mauritania |  |
| Cameroon | Mauritius |  |
| Cabo Verde | Morocco |  |
| Central Afr. Rep. | Namibia |  |
| Chad | Niger |  |
| Comoros | Nigeria |  |
| Congo, Dem. Rep. | Rwanda |  |
| Congo, Rep. | Senegal |  |
| Cote d'Ivoire | Sierra Leone |  |
| Equatorial Guinea | South Africa |  |
| Gabon | Sudan |  |
| The Gambia | Swaziland |  |
| Ghana | Tanzania |  |

**Note**: Note: WDI represents World Bank's World Development Indicators. WGI represents World Bank's Worldwide Governance Indicators. Countries were selected on the basis of data availability.
